# Low‐Field NMR Relaxometry for Elucidating the Effect of Temperature on the Rehydration Kinetics and pH and Ionic Strength on Rehydration Capacity of Texturized Soy Protein

**DOI:** 10.1002/mrc.70137

**Published:** 2026-07-21

**Authors:** Louise Margrethe Arildsen Jakobsen, Narges Fathali, Heidi Najbjerg, Hanne Christine Bertram

**Affiliations:** ^1^ Department of Food Science Aarhus University Aarhus Denmark

**Keywords:** low‐field NMR relaxometry, real‐time hydration monitoring, soaking conditions, structure–function modifications, texturized vegetable protein, water compartmentalization

## Abstract

Rehydration of texturized vegetable proteins (TVPs) is decisive for their functional properties, but little is known in relation to how rehydration conditions impact their ability to absorb and retain water. The present study aimed to investigate how rehydration dynamics and intrinsic water mobility of texturized soy protein are affected by temperature, pH, and ionic strength under dynamic or equilibrium conditions. Low‐field NMR T_2_ relaxation measurements were conducted dynamically on soy TVP during rehydration under different combinations of pH (5.4, 6.2, or 7.0), ionic strength (0.29, 0.46, or 0.71 M), and temperature (15, 25, or 35°C). Rehydration could be tracked from a decrease in the T_2_ relaxation time constant, reflecting a reduced mobility of water protons when water molecules were immobilized in intrinsic structures. Kinetic modeling revealed a significant effect of temperature on the rate of rehydration, whereas pH and ionic strength did not affect the rate of rehydration. To evaluate equilibrium conditions, 24 h soaking experiments were conducted, which revealed significant effects of pH on the rehydration capacity and NMR T_2_ relaxation. Increasing pH increased the rehydration capacity and T_2_ relaxation time constants, indicating that water compartmentalization in TVPs can be modulated through pH effects on electrostatic repulsion. In conclusion, NMR relaxometry enables kinetic studies of soy TVP rehydration, and this study demonstrates that rehydration kinetics and equilibrium rehydration capacity can be decoupled. Temperature primarily governed the rate of water uptake, whereas pH influenced final rehydration capacity, while ionic strength in the range applied here did not affect rehydration capacity.

## Graphical abstract

NMR T_2_ relaxation measurements enabled following hydration kinetics of texturized soy protein in real time, and to examine the effects of pH and ionic strength on water mobility in rehydrated, texturized soy protein at equilibrium.

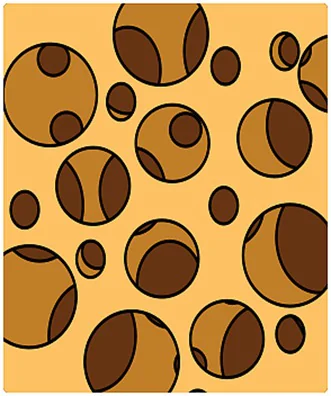

## 1 Introduction

In recent years, texturized vegetable proteins (TVPs) have emerged as a main ingredient used in plant‐based meat analogues. TVPs are manufactured by the application of an extrusion process, and moisture content can vary. Processing parameters, especially temperature, applied during extrusion impact the final product characteristics [1], but also pH can influence intrinsic structure formation [2]. As a result of high shelf stability, low‐moisture TVPs (LM‐TVP) are associated with ease of handling and storage [3]. Prior to further use, LM‐TVPs are hydrated and subsequently combined with other ingredients to obtain products such as plant‐based burgers and a variety of comminuted products such as meatballs.

The physicochemical and functional properties of TVPs are important for their attributes as ingredients and final product quality and consumer acceptability. Hydration capacity, i.e., the ability to absorb and retain water is one of the important functional properties. Thus, Hong et al. (2022) showed that hydration capacity was the most decisive factor affecting not only juiciness but also shear force and texture of patties prepared from rehydrated TVPs. A study on TVPs prepared from mixtures of soy, amaranth flour, sesame, and faba bean found that expansion ratio following hydration of the TVPs affected their texture profile [4]. However, only minor research has been conducted to understand how the ability to absorb and retain water of TVPs could be affected by rehydration conditions. In muscle meat, the ability of the myofibrillar proteins to swell and retain water is influenced by pH and ionic strength [5, 6]. Under pH conditions close to the isoelectric point (pI) of myosin (~5), the net electrostatic charges are low and repulsion therefore also low with accompanying small intermolecular spacing for water molecules. On the contrary, when pH is either lowered or increased compared with the pI, the net electrostatic charges increase and thereby also the electrostatic repulsion, [7]. which creates space for water molecules. In addition, the charge of the myofibrillar proteins is altered by ionic strength as the binding of negatively charged ions leads to a shift in pI [5]. Similar effects could be hypothesized in TVPs. Although effects of pH and ionic strength have been investigated in isolated plant proteins such as pea [8], to the best of our knowledge, no former studies have investigated how the rehydration behavior of TVPs might be optimized through varying pH and salt conditions. The aim of the present study was therefore to investigate how rehydration and intrinsic water mobility of TVPs are affected by temperature, pH and ionic strength under both dynamic and equilibrium conditions. For this purpose, low‐field NMR relaxometry, which is a non‐invasive and non‐destructive method that allows repeated and dynamic measurements [9] was applied. Rehydration studies were performed under two conditions: 1) normal soaking practice with continuous NMR measurements during 74 min of rehydration, and 2) overnight (24 h) soaking to ensure equilibrium and eliminate transient diffusion.

## 2 Materials and Methods

### 2.1 Chemicals and Sample Materials

Texturized soy (product name: Organic soy chunks) was purchased from a commercial supplier (KoRo Handels GmbH, Berlin, Germany). The texturized soy product contained 50% (w/w) protein, 8% (w/w) fat, and 29% (w/w) carbohydrates.

For the rehydration experiments, a total of nine buffers, which were combinations of three various pH values (pH 5.4, 6.2, 7.0) and three various ionic strengths (IS 0.29, 0.45, 0.71 M), prepared using 50 mM citric acid; H_2_O (10.507 g/L) and trisodiumcitrate; 2 H_2_O (14.707 g/L). pH was adjusted with 1 M NaOH or HCl and ionic strength adjusted with NaCl. The employed values of pH and ionic strength were adapted from previous studies on myofibrillar proteins [3].

### 2.2 Dynamic Real‐Time Rehydration Experiments

Preliminary tests showed that a ratio of 1:2 (w/w) was sufficient to avoid water limitation during rehydration. Therefore, one TVP chunk with a weight of approx. 0.25 g was put into an NMR tube with a diameter of 18 mm, buffer was added in a ratio of 1:2 (w/w), and the NMR tube was immediately inserted in the Bruker bench‐top mq20 NMR instrument (Bruker BioSpin GmbH, Rheinstetten, Germany) used for the measurements. The NMR instrument with a magnetic field strength of 0.47 T was equipped with an 18‐mm temperature variable probe and the temperature of the probe was controlled by an external cryo bath. Transverse (T_2_) relaxation measurements were conducted using the Carr–Purcell–Meiboom–Gill (CPMG) sequence at 154 s intervals (2.5 min) during a period of 74 min (1 h and 14 min), resulting in 30 measurements. A measuring period of 74 min was chosen to resemble the most dynamic period of hydration. Commonly applied rehydration conditions in the literature are within 5–10 min up to 2–3 h [10, 11, 12]. The acquisition parameters applied to acquire T_2_ relaxation curves included an interpulse spacing of 0.15 ms, 16 scans, a recycle delay of 5 s and 2000 echoes. The experiments were performed with a 90° pulse length of 11.58 μs and a 180° pulse length of 22.96 μs. During the measuring period, the sample tube remained within the NMR instrument. Experiments were conducted using three different measurement temperatures: 15, 25, and 35°C and including the three different combinations of buffer with varying pH (5.4, 6.2, and 7.0). For all combinations of temperature and buffer, measurements were conducted on three replicates from the same batch of texturized soy protein (*n* = 27).

### 2.3 Rehydration Kinetics Modeling

To assess the rehydration kinetics of TVP, non‐linear modeling was employed on the T_2_ relaxation time from the real‐time hydration experiment. The T_2_ relaxation decay was fitted to a mono‐exponential decay curve with an asymptote:

$$y=a*\exp^{-b*x}+c$$

where “a” expresses the initial T_2_ relaxation time immediately after initiating hydration, “b” is the decay rate of the hydration process indicating how quickly water is absorbed by the TVP and “c” corresponds to the equilibrium T_2_ relaxation time, representing the minimum water mobility reached at the end of the hydration process. Kinetic modeling was performed in R studio (version 2025.09.0, Posit Software, PBC, Boston, Massachusetts) using the nls() function with the SSasymp() self‐starting function. The model took the form y = Asym + (R0‐Asym) * exp(−exp (lrc) * x), where Asym represents the horizontal asymptote on the right side, R0 represents the response when input is zero and lrc is the natural logarithm of the rate constant. Parameter conversion was performed to achieve similar output as Ates et al. (2025) [13]: a = R0‐Asym, b = exp (lrc) and c = Asym.

### 2.4 Equilibrium Experiment

A static rehydration experiment was conducted, which entailed a rehydration time of 24 h to ensure equilibrium conditions. Approximately 1 g of dry TVP material was weighed and rehydrated in 50 mL buffer, and samples were placed at 4°C. After 24 h, samples were strained through a metal sieve and transferred to a 50 mL Falcon tube with a folded paper tissue in the bottom. Centrifugation was then performed at 3000 rpm for 10 min at 20°C (Megafuge ST4R Plus, ThermoFischer Scientific, Osterode am Harz, Germany).

The weight of the TVP sample was recorded before rehydration (W_before hydration), after soaking and after centrifugation (W_after centrifugation). The rehydration capacity (RHC) expressed in percentage was quantified as follows:

$$\mathrm{RHC}\%=\frac{W_{\text{after centrifugation}}\left(g\right)-W_{b\text{efore rehydration}}\left(g\right)}{W_{\text{before rehydration}}\left(g\right)}*100$$

Transverse (T_2_) relaxation was measured on the TVP samples following rehydration and draining using the same NMR instrument, the CPMG sequence and same acquisition parameters as described for the dynamic rehydration experiments. Multiple strategies were used to analyze the resulting T_2_ relaxation data. To obtain a continuous exponential distribution that allows for the identification of water populations such as those originating from different pore sizes, the Contin algorithm was applied using the build‐in program in the instrument software [14]. More quantitative T_2_‐components for statistics were obtained by discrete exponential fitting analysis performed using the EXPFIT algorithm developed by Pedersen et al. (2022) [12]. in MATLAB R2020b (version 9.9.0.1467703, The Mathworks Inc., Natick, Massachusetts). Bi‐exponential fitting was applied in the 24 h soaking experiments, where distinct populations were identified in the Contin analysis. In the dynamic experiments, mono‐exponential fitting was applied to account for the non‐equilibrium condition. For all nine buffers, three replicates were included (*n* = 27).

### 2.5 Statistical Analysis

Analysis of variance (ANOVA) was conducted in R studio (version 2025.09.0, Posit Software, PBC, Boston, Massachusetts) to determine significant main or interaction effects of pH, ionic strength, or temperature on NMR T_2_ relaxation time constants and magnitudes as well as hydration capacity. A significance level of 0.05 was applied. Prior to ANOVA, assumptions of homogeneity of variance and normality were checked using Levene's test and Shapiro–Wilk's test, respectively. If assumptions were not met, the Kruskal–Wallis non‐parametric test was used. Each measurement was conducted in triplicate. Pairwise comparisons of significant main effects were contrasted using Tukey HSD or Dunn's test (non‐parametric).

## 3 Results

### 3.1 Real‐Time Rehydration Kinetics Under Varying Temperatures

To investigate the rehydration kinetics of TVP, dynamic measurements were performed during rehydration of soy TVP at different temperatures. Figure 1 displays evolution in T_2_ relaxation time constants obtained from mono‐exponential fitting of T_2_ relaxation data acquired during dynamic measurements of rehydration at 15, 25, and 35°C, respectively. It was evident that the T_2_ relaxation time constants decreased over time from initial values of approx. 800 ms to approx. 100 ms at the end of the total soaking time of 74 min. The T_2_ relaxation time constants obtained as a function of rehydration time were fitted using mono‐exponential regression with an asymptote (Table 1). Statistical analysis revealed a significant effect of temperature on “b” (*p* = 0.003), corresponding to a faster reduction in the T_2_ relaxation time constants with higher temperatures reflecting a faster rate of absorption of water in the TVP structure with increasing temperature. The “b” values were significantly different for 15°C and 35 °C (*p* = 0.003), whereas the values were not significantly different between 15°C and 25°C (*p* = 0.17) and between 25°C and 35°C (*p* = 0.086). No significant effect of pH or Temp x pH interaction on “b” nor effects on constants “a” or “c” were found.

**FIGURE 1:**
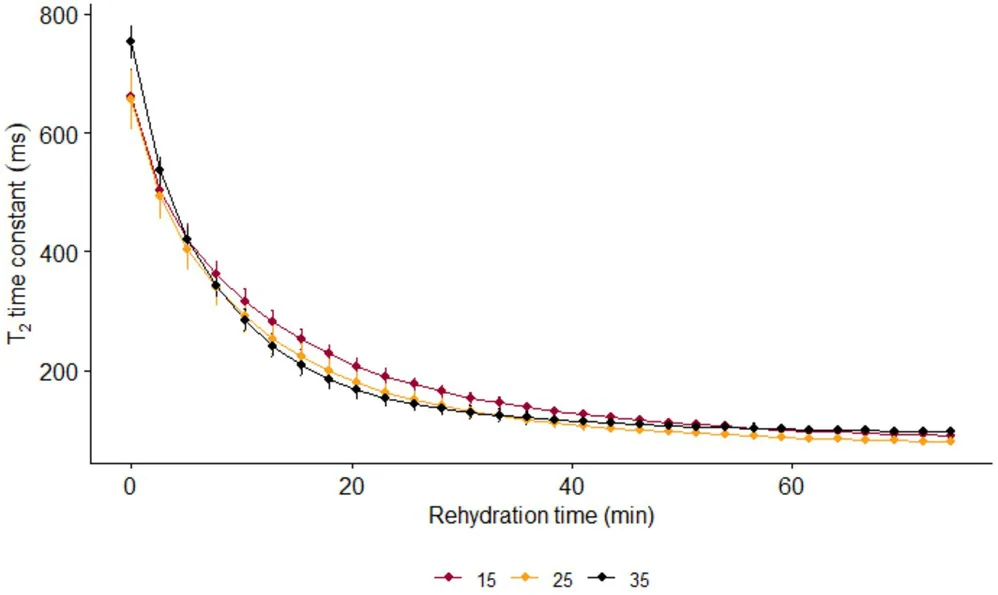
Evolution of T_2_ relaxation time constant during rehydration of TVP at three temperatures (15, 25, 35°C).

**TABLE 1:** Rehydration curve constants of TVP at different temperatures and pH values fitted on T_2_ relaxation time constants using mono‐exponential decay function with an asymptote, where “a”: initial T_2_ relaxation time immediately after initiating hydration, “b”: hydration process decay rate, and “c”: equilibrium T_2_ relaxation time. Values represent means ± SD, Kruskal–Wallis *p*‐values. Different superscript letters within the same column indicate significant differences as evaluated by Dunn's test using a significance level of 0.05.

Temperature	pH	a	b	c
15	5.4	498.07	0.09^a^	94.47
	6.2	474.58	0.07^a^	108.29
	7.0	600.32	0.08^a^	98.54
25	5.4	545.41	0.12^ab^	88.15
	6.2	475.60	0.10^ab^	87.43
	7.0	609.33	0.08^ab^	88.24
35	5.4	613.67	0.11^b^	108.14
	6.2	600.34	0.12^b^	116.68
	7.0	661.90	0.14^b^	93.62
*p*‐values				
Temperature		0.17	0.003	0.086
pH		0.23	0.80	0.87
Interaction		0.57	0.083	0.43

### 3.2 24 h Rehydration Studies

To investigate the hydration behavior of TVP at full rehydration, soy TVPs were soaked in buffer for 24 h at 4°C. This allowed assessing the rehydration capacity at equilibrium.

#### 3.2.1 Rehydration Capacity (RHC)

Figure 2 presents RHC of samples at different combinations of pH and ionic strengths. The RHC as expressed in percentage overall varied between approx. 150% to 180%. A two‐way ANOVA examining the effects of pH and ionic strength showed a significant effect of pH (*p* = 0.012) with an increase in RHC with increasing pH. A pH of 7.0 was associated with a significantly higher RHC compared to pH 5.4 (*p* = 0.012), and RHC was significantly higher for pH 6.2 compared with 5.4 (*p* = 0.012). There was no significant difference between 6.2 and 7.0 (*p* = 0.72). The main effect of IS was not significant (*p* = 0.47), and the pH × IS interaction effect was also not significant (*p* = 0.10).

**FIGURE 2:**
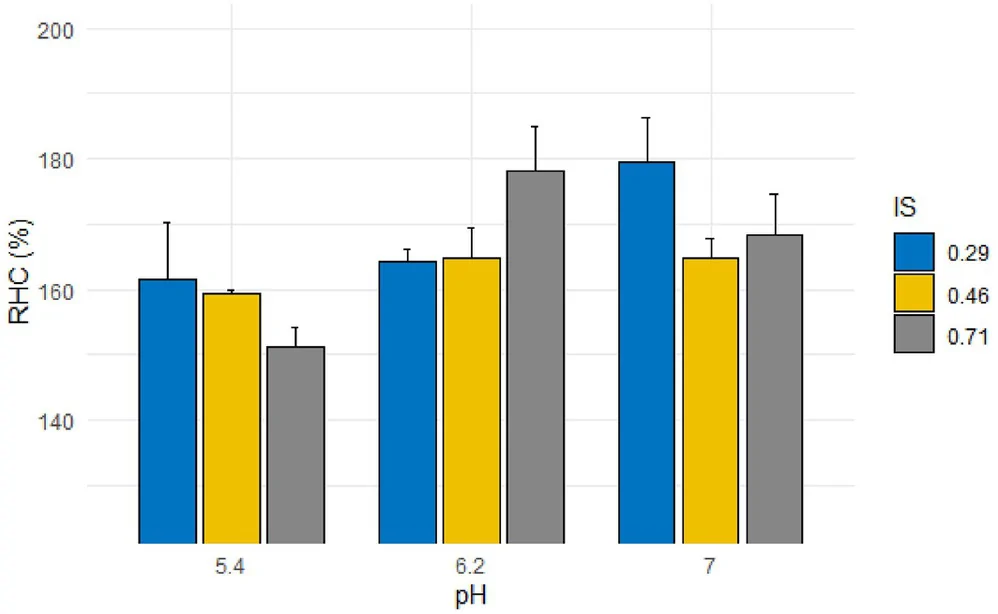
Rehydration capacity (RHC) (%) across pH levels for different ionic strengths (IS).

#### 3.2.2 NMR T_2_ Relaxation

Figure 3 shows distributed T_2_ relaxation times for the different combinations of pH and ionic strength measured after soaking for 24 h. The distributed T_2_ relaxation times showed a widespread distribution of T_2_ relaxation times but with a clear and major T_2_ population centered around 50–120 ms with an additional overlapping T_2_ population appearing as a shoulder on the right. It was evident that the major T_2_ population shifted toward longer relaxation times with increasing pH, and this effect was most evident at the lowest ionic strength of 0.29 M. For a more quantitative analysis, discrete bi‐exponential fitting was applied on the obtained T_2_ relaxation data (Figure 4a,b). Statistical analysis revealed an effect of pH on the shortest T_2_ relaxation time (T_21_ ~ 40–51 ms, *p* < 0.001) representing the major T_2_ population (Table 2). IS and the pH × IS interaction effects were not significant. At higher pH, the T_21_ time constant increased corresponding to a higher mobility of the water protons, the differences being significant between pH 6.2 and 5.4 (*p* < 0.0001), 5.4 and 7.0 (*p* < 0.0001), but not between 6.2 and 7.0 (*p* = 0.21). For the proportion of the two T_2_ populations, M1 and M2, M2 increased at the expense of M1 with increasing pH (Table 2). Pearsons's correlation analysis showed high correlation between RHC and T_21_ (Figure 5, R = 0.75, *p* < 0.001).

**FIGURE 3:**
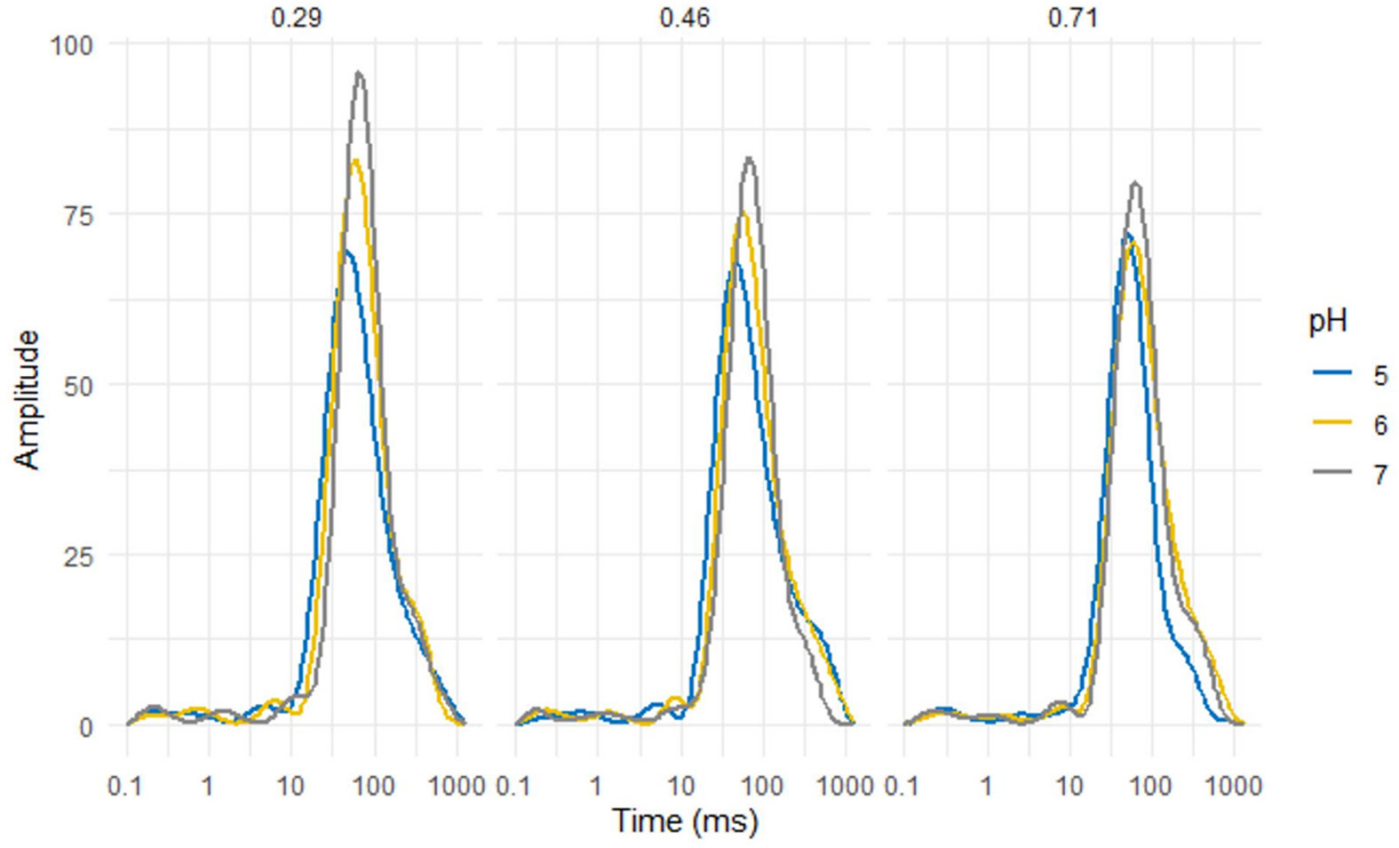
Distributed NMR T_2_ relaxation times as function of ionic strength and pH.

**FIGURE 4:**
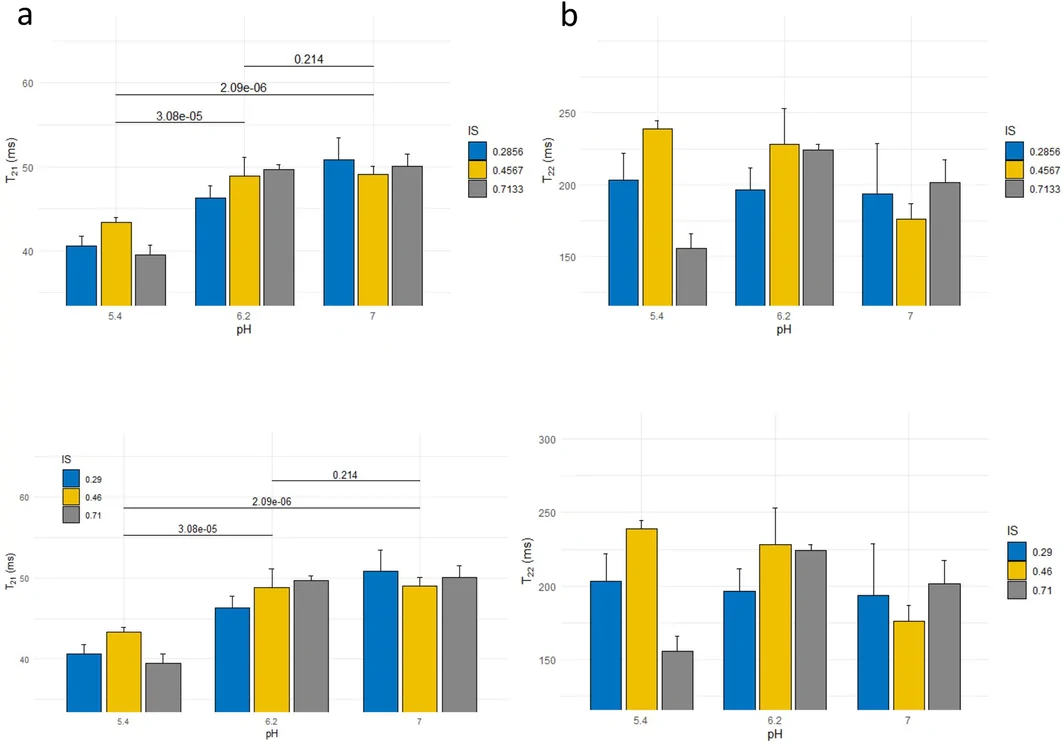
Discrete bi‐exponential fit of NMR T_2_ relaxation data a) T_21_ and b) T_22_.

**TABLE 2:** Discrete bi‐exponential fitting and rehydration capacity (RHC %) of data from 24 h soaking of TVP under different pH and ionic strength (IS) levels. Values represent means and *p*‐values are from ANOVA. Different superscript letters within same column indicate significant differences as evaluated by Tukey HSD post hoc test.

pH	IS	M1 (%)	M2 (%)	T_21_ (ms)	T_22_ (ms)	RHC (%)
5.4	0.29	69.35	30.65	40.58^a^	202.99	161.52^ab^
	0.46	69.50	30.50	43.36^ab^	238.63	159.29^ab^
	0.71	70.91	29.09	39.51^a^	155.81	151.30^a^
6.2	0.29	67.73	32.27	46.30^bc^	196.20	164.19^abc^
	0.46	68.18	31.82	48.86^cd^	228.37	164.87^abc^
	0.71	67.86	32.14	49.65^cd^	224.12	178.09^c^
7.0	0.29	66.30	33.70	50.87^d^	193.48	179.39^c^
	0.46	66.06	33.94	49.04^cd^	175.93	164.71^abc^
	0.71	68.50	31.50	50.06^cd^	201.69	168.36^bc^
*p*‐values						
pH		0.016	0.016	< 0.001	0.61	0.012
IS		0.24	0.24	0.80	0.72	0.71
Interaction		0.79	0.79	0.80	0.13	0.95

**FIGURE 5:**
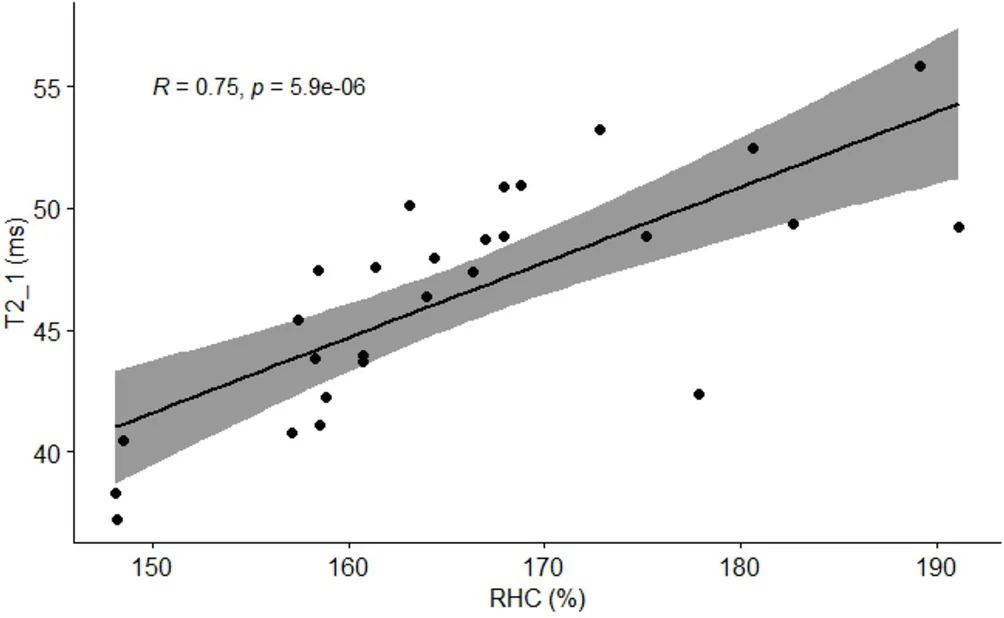
Pearsons's correlation between T_21_ time constant and rehydration capacity (RHC) (%) of TVP samples rehydrated for 24 h.

## 4 Discussion

### 4.1 Rehydration Kinetics Are Diffusion‐Limited and Temperature‐Dependent

An attractive attribute of low‐field NMR relaxometry is that it is non‐destructive and compatible with dynamic measurements. Ates et al. (2025) [10] recently demonstrated that low‐field NMR relaxometry can be employed for dynamic studies on the hydration of aqua faba powder. In the present study, we employed low‐field NMR relaxometry for real‐time monitoring of water immobilization during TVP rehydration. We found that pH and ionic strength had no effect on the rate of water immobilization in TVPs during the first hours of rehydration. However, temperature significantly affected the rate of water uptake. A higher temperature facilitated a faster water immobilization, which was reflected in a faster reduction in the T_2_ relaxation time, revealing that the rate of the rehydration process is higher at a higher temperature. This phenomenon probably occurs because a higher temperature results in faster movement of the water molecules and thus higher diffusion of water into the TVP and thereby an acceleration of water immobilization during the rehydration process.

### 4.2 T_2_ Relaxation Pattern After Rehydration of Texturized Soy Protein Is Governed by Proton Exchange and Structural Anisotropy

Rehydration of TVP has mostly been optimized empirically, and systematic studies on the rehydration process are sparse. Distributed NMR T_2_ relaxation times measured on texturized soy after 24 h rehydration revealed a continuum of T_2_ relaxation times with a major population centered around 40–50 ms (T_21_) and an overlapping population centered around 200 ms (T_22_) (Figure 3). In a study on high moisture soy protein extrudates, two mobile T_2_ populations with relaxation times around 18 and 75 ms were reported after rehydration [15], which is comparable with the two mobile T_2_ populations found in the present study on low moisture texturized soy protein. Kuipers et al. (2024) [13] ascribed the two mobile T_2_ populations to a protein‐rich and a water‐rich phase, respectively, and by comparison with T_1_ relaxation times they further concluded that the two populations are governed by the same proton exchange mechanisms while the presence of two water populations can be ascribed to structural heterogeneity and absence of diffusional averaging. Consistent with this assignment, we anticipate that the presence of the two slow‐relaxing T_2_ populations (T_21_, T_22_) can be ascribed to intrinsic water within confined protein structures that experience anisotropy, whereas the fast‐relaxing T_2_ populations in the ~0.1–1 ms range originate from protons in macromolecules.

### 4.3 Rehydration Capacity (RHC) Is Modifiable Through Electrostatic Swelling

The present study investigated how pH and ionic strength influenced the rehydration of soy TVP. NMR relaxation measurements were conducted after 24 h of rehydration where equilibrium is anticipated. Even though it has been shown that rehydration temperature affects structure and thereby texture of TVPs [16], the 24 h rehydration studies were not conducted at the same temperatures as the dynamic measurements but at 4°C to establish practical use conditions. Contrary to the dynamic studies, we found a significant effect of pH on both RHC and on the NMR T_2_ relaxation after 24 h where equilibrium is anticipated. Thus, at pH 6.2 and 7.0 RHC increased corresponding to a higher extent of water immobilization, and concomitantly a longer T_21_ relaxation time constant was observed compared with pH 5.4. TVPs have a network of pores and channels within their fibrous structure that are held together by disulfide cross‐links in the proteins. In the presence of water, the liquid enters these pores and is held there by capillary action. In porous media, longer T_2_ relaxation times reflect larger pore sizes according to the Brownstein–Tarr model [17]. Thus, the present findings suggest that the sizes of intrinsic pores in TVPs can be modulated through effects of pH on electrostatic repulsion. The strong positive correlation between RHC and the T_21_ relaxation time constant (Figure 5) can be attributed to the associations between electrostatic repulsion, pore sizes and thereby amount of water that can be retained within the intrinsic protein structures. In other high‐moisture foods, a longer T_2_ relaxation time is also consistent with a higher swelling and pore size [18, 19]. In muscle‐based systems, pH and ionic strength have great influence on water‐holding capacity, which can be attributed to the myofibrillar proteins and changes in electrostatic repulsion impacting the interfibrillar spaces. Thus, on isolated myofibrillar proteins, it has been shown that water reuptake and intrinsic water mobility as reflected in T_2_ relaxation times are strongly influenced by pH and ionic strength [5].

Soy protein mainly consists of the globulins β‐conglycinin (7S) and glycinin (11S). During extrusion processing proteins are unfolded and may undergo intermolecular interactions, including disulfide bond formation. Bases on previous studies, glycinin (11S), containing a high number of sulfide‐containing amino acids, has been proposed to contribute to texturization through covalent cross‐linking and protein aggregation, thereby reducing solubility [20]. Thus, it could be hypothesized that the covalent crosslinking from texturization will create structural constraints for swelling through electrostatic repulsion effects. However, the NMR T_2_ relaxation data together with data on RHC in the present study suggest that swelling of soy TVPs can occur through electrostatic repulsion effects. Even if extrusion promotes protein aggregation, charged amino acid residues may remain accessible at the protein surface, allowing electrostatic interactions to influence hydration behavior. Samard et al. (2019) [19] showed that extrusion generally reduces the protein solubility of plant proteins, but a pH effect below and above pH 4 remains (0.1 M NaCl), indicating that electrostatic repulsion effects remain after extrusion. It is also possible that β‐conglycinin (7S), which has a low content of sulfur‐containing amino acids, contribute to non‐covalent binding and remains soluble thus contributing to repulsion, while 11S is more cross‐linked and limits spatial expansion. The present study did not include fractionation or compositional analysis of the soy TVP and thus the relative contribution of 7S and 11S proteins cannot be determined directly. However, the interpretation is consistent with Fie et al. (2025) [21], who showed that soybean protein isolates with higher 7S/11S ratio correlated with higher water‐holding capacity. Although the present study demonstrates resembling effects of soy TVP hydration behavior, the underlying contribution to specific protein fractions was not investigated directly. A protein surface becomes negatively charged at pH levels above the isoelectric point (pI). Soy protein isolates have a pI around pH 4.5 [22]. Accordingly, in the present study it could be expected that lowest surface charge of the proteins would be at pH 5.4 and highest surface charge of the proteins would be at pH 7.0. The results suggest that these changes in surface charge of the proteins and electrostatic repulsion did influence intrinsic structures and RHC of TVPs. Thus, by modifying pH of the liquid used for rehydration of TVPs, a higher extent of hydration can be achieved through increased electrostatic repulsion and pore expansion. The results are coherent with Wang et al. (2009) [22], who found that the highest water resistance of powders of soy protein isolates was obtained a neutral surface charge of the protein at the pI.

Other investigations on effects of pH and ionic strength modifications on TVPs are sparse. Maung et al. [23]. studied how fermentation with *Bacillus subtilis* prior to extrusion affected the textural characteristics of TVPs. *B. subtilis* fermentation resulted in a minor pH decrease, from approx. pH 7 to pH 6.5. This minor pH decrease was accompanied by structural changes in the TVP as the protein network was diminished following fermentation. Since the fermentation probably also resulted in proteolysis, the conditions were different from the present study where we exclusively studied effects of pH during rehydration.

Proteins can carry both negative and positive charges depending on pH. Variations in ionic strength can therefore affect electrostatic repulsion and thereby also intrinsic pore sizes and water retention capacity. A recent study on pea protein isolate showed that increasing the NaCl concentrations from 0.1 to 0.4 M significantly increased protein solubility at pH 7 [24]. However, in the present study where we investigated a span in ionic strength from 0.29 M to 0.71 M, we did not observe any effect of ionic strength. We speculate whether this could be due to the ionic strength tested that were too high for soy proteins and that the surface charge is already neutralized at 0.29 M and that different effects would have been evident at lower ionic strength. Lee et al. (2003) [22] showed that protein solubility of different soy protein isolates was most affected at ionic strengths below 0.25 M NaCl at pH 7.0 and that above this level, protein solubility was consistently low, confirming a salting‐out effect. These findings suggest that within 0.29–0.71 M, IS does not modulate electrostatic repulsion and RHC of soy TVP. Future studies on soy TVP rehydration should focus on IS in the 0–0.29 M range to confirm the mechanisms suggested by Lee et al. (2003) on isolates.

## 5 Conclusion

In the present study we investigated the effect of temperature, pH and ionic strength on rehydration kinetics and capacity of soy TVP using NMR relaxometry. Using dynamic measurements, we demonstrated that NMR relaxometry is a useful method for evaluating the local pore environment as the T_2_ relaxation time constant decreases as free water is absorbed and immobilized in the TVP structures by capillary forces. In addition, we showed that temperature exhibited a significant effect on the kinetics of hydration of soy TVPs while no effects of pH or ionic strength were observed. However, under equilibrium conditions after 24 h of soaking, the rehydration capacity of soy TVP was pH‐dependent, showing highest capacity at pH 7.0, which likely could be attributed to increased electrostatic repulsion when pH increased from pH 5.4 to 7.0. Within a range of 0.29 to 0.71 M, ionic strength did not impact rehydration capacity of the soy TVPs. The effect of modifying ionic strength below 0.29 M and above 0.71 M on soy TVP rehydration capacity remains to be established. In conclusion, NMR relaxometry revealed that temperature is the major driver of rehydration kinetics, while under equilibrium conditions, pH is the major lever for maximizing soy TVP rehydration capacity.

## Author Contributions

**Louise Margrethe Arildsen Jakobsen:** conceptualization, writing – original draft, methodology, software, visualization, data curation, formal analysis, writing – review and editing. **Narges Fathali:** investigation, writing – review and editing. **Heidi Najbjerg:** investigation, writing – review and editing. **Hanne Christine Bertram:** writing – review and editing, writing – original draft, funding acquisition, conceptualization, project administration, supervision, resources.

## Funding

This study was funded by Aarhus University Research Foundation (project no. AUFF‐E‐2023‐9‐58 “CHEW‐NMR”).

## Conflicts of Interest

The authors declare no conflicts of interest.

## Data Availability

The data that support the findings of this study are available from the corresponding author upon reasonable request.
